# Diversity of Mercury-Tolerant Microorganisms

**DOI:** 10.3390/microorganisms13061350

**Published:** 2025-06-10

**Authors:** Anastasia A. Golysheva, Lyudmila V. Litvinenko, Irina B. Ivshina

**Affiliations:** 1 Institute of Ecology and Genetics of Microorganisms, Perm Federal Research Center of the Ural Branch of the Russian Academy of Sciences, 13 Golev Str., Perm 614081, Russia; 2 Department of Microbiology and Immunology, Perm State National Research University, 15 Bukirev Street, Perm 614068, Russia

**Keywords:** microorganisms, heavy metals, mercury, mercury tolerance, bioremediation

## Abstract

Researchers have identified mercury as one of the most toxic environmental pollutants, with deleterious effects on human health and biota. Microorganisms play a key role in the accumulation, degradation, and neutralisation of mercury. Numerous bacteria, fungi, and microalgae possess the mer operon and its homologues, which contain genes responsible for the transport and detoxification of mercury compounds. Mercury-tolerant Microorganisms efficiently convert mercury into less toxic forms. Their tolerance characteristics position them as promising agents for the remediation of ecosystems altered by human activity. This review explores the mechanisms by which microorganisms resist mercury and their potential for biotechnological applications, including eco-friendly and cost-effective bioremediation of mercury-contaminated environments.

## 1. Introduction

According to WHO, mercury (Hg) is among the top ten most hazardous heavy metals (HM) and belongs to a group of six highly hazardous HMs, which include arsenic (As), cadmium (Cd), selenium (Se), lead (Pb), zinc (Zn), and mercury (Hg) itself. The annual intake of Hg compounds from anthropogenic sources near industrial facilities exceeds natural background levels several times [1].

A plethora of chemical and physical technologies, including adsorption, chemical precipitation, ion exchange, electrochemical treatment, membrane filtration, and reverse osmosis, are employed for the removal of metals from industrial effluents. Nevertheless, these processes are inefficient and economically disadvantageous compared to biological treatment methods [2,3,4]. Bioaccumulation and biosorption processes, based on the use of microorganisms and their enzymatic activity, are recognised as the most promising [5,6,7]. It is universally acknowledged that bioremediation is an effective and environmentally safe method for the remediation of environments contaminated with highly toxic HMs [8].

As with other transition metals, mercury is neither naturally degradable nor biodegradable, which makes its removal from the environment difficult. The restoration of Hg-contaminated ecosystems relies on processes that directly remove Hg, immobilise it, or transform it into harmless forms [9].

It is evident that mercury compounds, even in minute quantities, pose a threat to human health and life. These compounds have been shown to cause damage to the central nervous system, as well as to the respiratory and cardiovascular systems, and to induce DNA mutations, which can ultimately lead to the development of cancerous tumours. The toxicity of mercury is attributable to its capacity to bind to sulfhydryl or disulfide groups in proteins, resulting in the inactivation of enzymes. The clinical manifestations of mercury poisoning, also known as mercurialism, encompass a wide spectrum of symptoms, including irritability, memory impairment, depression, weight loss, fatigue, paranoia, hallucinations, and impaired concentration and attention [10,11,12,13,14]. Furthermore, the bioaccumulation and biomagnification of organic Hg compounds through food webs pose an increased risk to living organisms [15].

Hg readily replaces iron in blood components, such as haemoglobin and other iron-containing molecules, forming strong chemical bonds that may lead to various pathological disorders [16]. In domestic settings, the primary route of human exposure to Hg are through its vapours, while contaminated food and drinking water constitute less significant sources [17].

To mitigate the toxic effects of Hg on living organisms, governments of many countries worldwide have promulgated legislative acts regulating the extraction, treatment, and transport of this hazardous substance. The primary instruments in this regard are the Basel Convention on the Control of Transboundary Movements of Hazardous Wastes and Their Disposal [18] and the Minamata Convention on Mercury [19].

Anthropogenic emissions of mercury into the atmosphere have reached 2.500 tons per year and continue to increase steadily [20]. Notably, current Hg concentrations in the atmosphere are 5.5 to 7.6 times higher than natural background levels [21,22]. Mercury exists in three distinct forms in the environment: Elemental (Hg^0^), inorganic (Hg^1+^ and Hg^2+^), and organic (MeHg, CH_3_Hg^+^, and CH_3_HgCH_3_). Inorganic mercury is ubiquitous in the natural environment, predominantly present in the form of Hg^2+^. However, reliable data concerning the content of Hg in the form of Hg^1+^ in the environment are very limited due to the chemical properties of the substance [23]. In aquatic and anaerobic environments, inorganic mercury frequently undergoes methylation, resulting in the formation of the organic form, which is the most toxic to living organisms. Organic mercury exists in two main forms: Monomethyl mercury (MeHg, CH_3_Hg⁺) and dimethyl mercury (CH_3_HgCH_3_). Monomethyl mercury is the most toxic, acting as a potent neurotoxin [24,25]. Elemental mercury (Hg⁰), the least toxic form, can exist as both a liquid and a gas at room temperature and can evaporate from surface water and soil [26].

The primary objective of this review is to investigate the potential of employing microorganisms to inactivate mercury contamination.

## 2. Sources of Environmental Inputs and Geochemical Cycling of Mercury

The main sources of Hg entering the biosphere are divided into two categories: Natural and anthropogenic. Natural sources include the entire Earth’s crust, rocks, and the surface of the world’s oceans, all containing dispersed Hg compounds. A separate category includes Hg released into the atmosphere during volcanic eruptions [27,28].

Anthropogenic sources of Hg entering the environment can be categorised into three main classes: First, current emissions resulting from the mobilisation of Hg impurities and compounds in raw materials; second, emissions during technological and manufacturing processes; and third, historical emissions, where Hg is released through remobilisation [29]. Historical emissions refer to mercury that was released into the environment in the past and is now re-entering active biogeochemical cycles due to changing conditions (e.g., soil erosion, climate change, economic activities), leading to renewed contamination of the environment. The predominant source of anthropogenic Hg emissions worldwide is fossil fuel combustion [30,31]. The extraction of precious metals also plays a significant role; the mercury amalgamation method is still used in gold mining [27,32]. The predominant form of emissions is Hg^0^, accounting for more than 90% of the total mercury in the atmosphere [33]. Hg^2+^ constitutes the majority of mercury in soils, where it is bound to organic compounds, clay, and sulfides [27,34].

The majority of Hg found in the environment is present in the form of inorganic and organomercury compounds, with the exception of atmospheric mercury. The most prevalent compounds are HgCl_2_, HgOH, and HgS. In addition, CH_3_HgCl and CH_3_HgOH are the main organomercury compounds, which, together with others, occur in negligible amounts [35].

The geochemical cycle of mercury plays a key role in providing mercury forms that serve as substrates for microorganisms capable of methylating mercury and converting it into organic compounds such as methylmercury [36]. The cycle involves the anaerobic oxidation of mercury, followed by the incorporation of dissolved Hg²⁺ into the system, a process necessary for the formation of MeHg. The elimination of Hg^0^ during the reduction process leads to a restricted synthesis of MeHg, attributable to the exhaustion of inorganic mercury substrates [37]. The pathways of mercury reduction in the oxygen-free zones, where the methylation process occurs, have been mainly characterised in laboratory experiments and remain the most difficult to evaluate in the field [38].

## 3. Microorganisms as Primary Responders to Exposure to Metallic Eco-Pollutants

### 3.1. Diversity of Mercury-Tolerant Microorganisms

Microorganisms (fungi, microalgae, and bacteria) have great potential to remove metal contaminants by biosorption, bioaccumulation, and biotransformation [39,40,41]. The primary benefits of bioremediation methods include the reduction of secondary contamination, the generation of economic benefits, and the potential for application to heavily HM-contaminated sites [42].

The cycling and transformation of HM in the environment are significantly influenced by microorganisms. In comparison to animals and plants, microorganisms demonstrate heightened sensitivity to metal stress [43,44].

It is well established that HMs, including Hg, are capable of inducing substantial morphological distortions in microbial cells, in addition to exerting an influence on the protein synthesis, lipid metabolism, respiration, and energy metabolism in microorganisms [45,46].

Intensive research into effective methods for preventing mercury contamination began in the 1950s, following the Minamata disaster in 1956. These measures became increasingly widespread as the consequences of the extensive use of fungicides and disinfectants containing organic mercury compounds became more apparent [47,48].

Researchers have shown a marked increase in interest in microorganisms that bioaccumulate mercury recently. Table 1 provides data on the source of isolation and taxonomic affiliation of some microorganisms with the ability to accumulate Hg.

The microorganisms studied so far play important roles in mercury cycling and have potential for bioremediation due to their detoxification abilities. However, these represent only a small fraction (Table 1) of the wide diversity of microorganisms investigated in this field. A broad range of microbial groups, differing in physiology and resistance mechanisms, are being studied to better understand how mercury resistance develops and functions across various environments. This diversity highlights the complexity of mercury contamination and the extensive scope of ongoing research aimed at harnessing microbial potential for environmental remediation.

### 3.2. Mechanisms of Tolerance of Microorganisms to Heavy Metals

In the field of microbiology, there is a growing understanding of the various strategies employed by microorganisms to increase resistance to toxic heavy metals. The most common mechanisms include [79]:

- The synthesis of organic acids and polysaccharides with chelating properties, capable of binding metal ions.
- The process of biosorption occurs at the level of cell walls of microorganisms.
- The intracellular accumulation of metals enables microorganisms to sequester toxic substances.
- The use of cysteine-rich buffer proteins, as these can bind to pollutants and reduce their toxicity.
- The sequestration in vacuoles is an effective method of storing mercury in an inactive metallic form.
- The chemical transformation of metals into less toxic forms.

These mechanisms enable microorganisms to survive in conditions of increased concentrations of toxic HMs, including Hg, and to adapt to extreme habitats. Exposure to low doses of negative factors or harmful substances can trigger the hormesis effect. This phenomenon is defined as the adaptive reactions of organisms to moderate environmental problems, thereby improving their functionality and/or increasing their ability to adapt to more serious influences in the future [80].

**Microalgae**. Microalgae are highly adaptable microorganisms capable of thriving in rapidly changing environmental conditions. Due to their rapid growth rates and biomass accumulation, microalgae are increasingly used in the development of methods for wastewater treatment targeting ecotoxicants [81].

Algae are characterised by several main mechanisms of tolerance to heavy metals (Table 2). The initial process is passive extracellular adsorption, or biosorption on the cell surface. The subsequent process is active intracellular accumulation, bioaccumulation, and biotransformation.

Microalgae have a high surface area-to-volume ratio, which facilitates HMs binding and accumulation. Passive processes occur through ion exchange, chelation, complexation, redox interactions, biomineralisation, and the deposition of insoluble complexes on their cell walls, extracellular polymeric substances, and intracellular compartments. The primary mechanisms encompass electrostatic, van der Waals, and hydrophobic interactions between the positively charged heavy metal cations and the negatively charged groups on the cell surface [82,83]. The process of biosorption is contingent upon a number of environmental factors, including temperature, solution pH, HM species, the presence of other ions in solution, and biomass [84]. The microorganism can actively take up metals through specific transport mechanisms. This process is accompanied by antioxidant reactions and changes in the redox state of cells [85].

The presence of HMs, including mercury, in aquatic environments can induce microalgae to produce elevated levels of reactive oxygen species (ROS). Excessive ROS generation in microalgal cells causes oxidative stress, leading to damage of vital cellular components such as proteins, lipids, and DNA. This oxidative damage disrupts the photosynthetic apparatus, reduces the activity of key enzymes involved in metabolism, and impairs cell division processes. As a result, the growth, photosynthetic efficiency, and overall viability of microalgae are negatively affected, which can alter their ecological roles and bioremediation potential [86,87].

**Fungi**. Microscopic fungi are widespread and ecologically important reductants and symbionts in soil and aquatic ecosystems, largely due to their diverse metabolic potential and versatile enzyme systems. In the context of mercury contamination, fungi play a significant role in mercury transformation and detoxification. Their intracellular and extracellular enzymes enable them to transform toxic mercury compounds into less harmful forms through processes such as methylation, demethylation, reduction, and biosorption. These mechanisms allow fungi to immobilize mercury, reduce its bioavailability, and mitigate its toxicity in the environment. Moreover, fungi can form symbiotic relationships with plants, enhancing phytoremediation efficiency by facilitating mercury uptake and tolerance. The ability of fungi to survive and function in mercury-contaminated environments makes them valuable agents for bioremediation strategies aimed at detoxifying mercury-polluted soils and waters [88,89].

One of the earliest cellular responses of microscopic fungi to HM is the generation of ROS and reactive nitrogen species (RNS), which lead to oxidative and/or nitrosative stress and disruption of the redox balance of the cell. Consequently, the antioxidant system, comprising compounds such as peroxidase, superoxide dismutase, and catalase, is activated [90].

The mechanisms of HM detoxification in fungi are categorised into two distinct types. The first involves the secretion of chemical compounds outside the cell to bind the metal in the extracellular space or on the cell wall, rendering it less toxic to the organism. The second type of detoxification is dominated by the chelation of toxic ions in the cytosol, which leads to inactivation and sequestration of heavy metals away from ongoing metabolic processes [91,92].

The bioaccumulation of HM by fungal cells is a complex process involving the interaction with organic acids and polymers, as well as their subsequent transportation to specific intracellular compartments. Cell wall ruptures resulting from metal exposure due to increased membrane permeability cause intracellular uptake and binding [90].

**Bacteria**. In bacteria, the principal mechanisms of protection and adaptation to HM are realised extracellularly due to alterations in pH and redox potential of the medium, mobilisation of phosphates, or production of polysaccharides, siderophores, and various antioxidant enzymes. Bacterial cells exhibit nonspecific resistance to HM and Hg ions through several known mechanisms. These mechanisms include (1) the presence of an extracellular barrier, (2) the active transport of metal ions out of the cell through ion channels, (3) the binding of biomolecules to the cell surface extracellularly, (4) the binding of biomolecules intracellularly, (5) the reduction of metal ions, and (6) the formation of a biofilm [79,93]. Concurrently, it is notable that a bacterial strain may possess multiple defence mechanisms in a concurrent manner.

The role of bacterial extracellular polymeric substances (EPS), particularly lipopolysaccharides (LPS) and exopolysaccharides (EPSs), in conjunction with environmental response mechanisms and quorum sensing signals, has been demonstrated to be significant in stress survival, bacterial aggregation, and the progression of biofilm development and colonisation. As asserted by Nocelli et al. (2016), nitrogen-fixing species of *Sinorhizobium* sp. have the capacity to produce EPS, succinoglycan, and galactoglucan, which contribute to their survival under HgCl_2_ contamination. Furthermore, co-cultivation of EPSs-producing and metal-tolerant strains has been shown to have a protective effect on Hg-sensitive strains that do not produce EPSs [94].

It can be posited that EPS fulfils a protective function in relation to bacterial cells, acting as a barrier against external exposure to HM. These substances have the capacity to bind with toxic ions outside the cells, thereby mitigating the adverse effects of metal cations. Phytoplankton have been demonstrated to influence the chemical composition of trace amounts of Hg^2+^ in the natural environment through reactions and have been observed to directly absorb Hg^2+^ through complexation of EPS with Hg^2+^. In the cyanobacterium *Microcystis aeruginosa*, there is the potential for cell walls, capsules, and extracellular products (carbohydrates, proteins) to interact with Hg^2+^ ions, which contributes to an increase in cellular tolerance to Hg [95,96].

**Table 2:** Mechanisms of resistance of microorganisms to HM and mercury.

Mechanisms of Resistance	Reference
**Microalgae**	
Metals are bound and sequestered by cell wall components (e.g., polysaccharides and proteins), and complexes are formed with HM on the cell surface	[97]
The expression of specific HM-binding peptides (phytochelatins and metallothioneins) results in the subsequent formation of peptide-HM complexes (phytochelatins and metallothioneins). For instance, phytochelatins form complexes with Hg^2+^.	[98]
Chelation of HM by organic acids, amino acids, and peptides with further formation of insoluble complexes	[99]
The synthesis of antioxidant enzymes in response to the presence of reactive oxygen species, including superoxide dismutase, peroxidase, and catalase	[100]
The synthesis and activation of non-enzymatic systems, including carotenoids, tocopherol, and glutathione	[101]
Overexpression of heat-shock proteins	[102]
Expression of genes encoding cation efflux proteins and HM ATPases, including mercury	[103,104]
**Fungi**	
The synthesis of chitin and chitosan with amino and hydroxyl groups for the purpose of biosorbing phenolic compounds, dyes, and heavy metals	[105]
Decrease in hyphae length and number of branches in response to metal stress. Changes in the distribution of fungal biomass within colonies	[106]
Excessive accumulation of hydrolase and oxidase; inhibition of colony growth	[107]
Changes in pigment synthesis	[108,109]
Synthesis of antioxidant enzymes in response to the presence of reactive oxygen species: Peroxidase, catalase, lignin peroxidase, manganese peroxidase	[110,111]
Increased production of HM-dependent ROS and RNS; modifications of RNA, DNA, and protein pools	[110]
Biosynthesis of organic acids in response to the presence of HM in the medium, e.g., oxalic, citric, succinic, malic, acetic, and gluconic acids for subsequent binding to HM ions	[112,113]
The synthesis of siderophores, which form complexes with HMs such as Cd, copper, Pb, Zn, nickel, and As, prevents these metals from being taken up by the cell	[114,115]
The interaction of HM ions with functional groups on the cell surface, including hydroxyl, amide, carboxyl, and phosphate groups	[116]
Synthesis of transporters of the ATP-binding cassette (ABC) family involved in intracellular HM transport	[117]
Synthesis of specific metal-binding proteins: Glutathione, phytochelatins, and metallothioneins	[118,119,120]
Active synthesis of transport proteins in the presence of excessive HM concentration in the medium, transport of HM ions from cytosol to vacuole to prevent toxicity	[121]
**Bacteria**	
Metal-tolerant strains precipitate HM ions (Pb^2+^, Hg^2+^, Cd^2+^, etc.) in the form of sulphide granules on the outer surface of cells	[5,122,123]
Use of HM ions as thermal acceptors in energy metabolism during anaerobic respiration: Reduction of Fe^3+^ to Fe^2+^, reduction of Cr^6+^ to Cr^3+^	[124]
Synthesis of surfactants of biological origin	[123]
Biofilm formation in response to metal stress. Synthesis of polymeric compounds to bind metal ions and prevent their entry into the complex and cells	[125]
Regulation of cell wall fluidity and permeability in response to changes in external conditions depending on the culture medium, the presence of toxic compounds in the medium and various stresses	[8,126]
Bioaccumulation of HM inside the cell through channel systems	[127]
Synthesis of siderophores—low molecular weight chelating compounds	[128,129]

It is evident that soil microorganisms demonstrate a high level of tolerance to HM. This tolerance is likely the result of evolutionary adaptation to contaminated environments.

### 3.3. Mechanisms of Bacterial Cell Resistance to Mercury

To date, research has focused on the adaptation mechanisms exhibited by bacteria in response to Hg exposure. Hg-tolerant bacteria are responsible for three major biological transformations of Hg: (1) Reduction of Hg^2+^ to metallic Hg^0^, (2) methylation of Hg^2+^ to MeHg, and (3) demethylation of MeHg to CH_4_ and Hg^2+^ with further volatilization of Hg^0^ [130].

#### 3.3.1. Nonspecific Resistance

**Figure 1:**
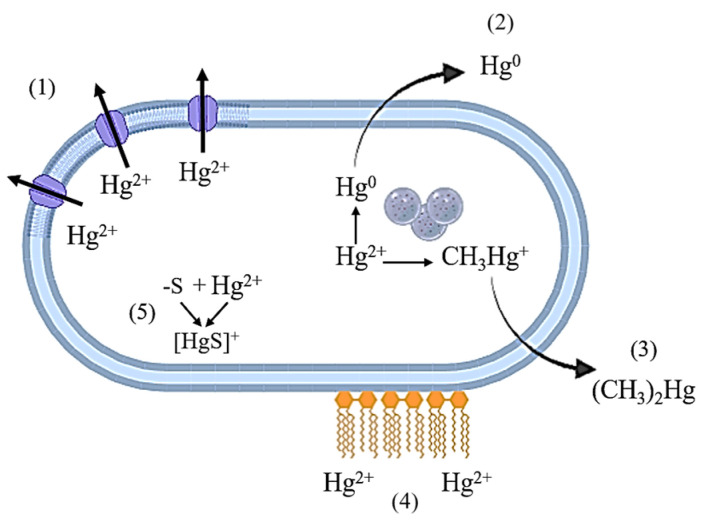
Mechanisms of resistance of bacterial cells to mercury compounds. 1—transport of Hg^2+^ ions from the cell through ion channels; 2—reduction to elemental Hg^0^; 3—methylation to volatile compounds; 4—extracellular binding to biomolecules on the cell surface; 5—formation of insoluble compounds with subsequent deposition in the cell.

The cellular mechanisms of resistance to Hg most often include (Figure 1) a decrease in the uptake of mercury ions from the medium, transport of mercury ions out of the cell, extracellular sequestration, and bioaccumulation [29]. The presence of unique physiological properties in certain groups of mercury-resistant bacteria increases their bioremediation potential.

The antioxidant system of the organism, especially in bacteria lacking the Hg resistance operon, is also involved in reducing the toxic effects of Hg on bacterial cells [131,132]. Gram-negative anaerobic bacteria *Shewanella oneidensis* demonstrate heightened sensitivity to Hg^2+^ ions under aerobic conditions compared to those involving fumarate reduction. The sensitivity of aerobic cells to mercury is attributable to metallic damage to the cell membrane rather than to damage of intracellular components. In this instance, mercury enters the cell and is deactivated by binding to glutathione. Under aerobic conditions, less mercury enters the cell, glutathione content is not reduced, and lipids are damaged [132]. This supports the notion that the components most sensitive to mercury may not be intracellular targets but rather macromolecules located on the cell membrane.

Bioremediation, in the context of heavy metal exposure to microbial cells, involves utilizing the ability of microorganisms to survive, transform, and neutralize toxic metals to remediate contaminated ecosystems. The process of bioremediation occurs through various mechanisms, including:

- *Redox processes and alkylation*. The process of bacterial methylation of Hg is contingent upon a number of environmental factors that affect metal bioavailability and microbial community structure. These factors include temperature, pH, redox potential, availability of nutrients and electron acceptors, as well as the presence of ligands and adsorbing surfaces [133]. In numerous systems, sulfate-reducing bacteria [66,67,134,135,136] and iron-reducing bacteria [137,138] have been observed to act as microbiological methylators of mercury. The biochemical mechanism of mercury methylation by these bacteria involves two pathways: One acetyl-CoA–dependent and one independent [139].
- *Passive adsorption*. The adsorption process is not contingent upon the metabolism of the bacterial cell. The metals are located on the cell surface as a consequence of electrostatic interactions, van der Waals forces, covalent binding, changes in the redox potential, or a combination of these processes. The mechanism in question has been explained by a number of processes, including precipitation and surface complexation, ion exchange as the sole dominant role, and physical adsorption [140]. According to the findings of François et al. (2012) [141], in the presence of HgCl_2_, inactivated bacterial biomass has been shown to remove between 40.0 and 120.0 mg Hg per g biomass dry weight while concurrently forming extracellular Hg deposits.

As previously referenced, bacteria have the capacity to produce EPS that prevents the toxic effects of Hg^2+^ on the cell [94,95,96]. Furthermore, the uptake of mercury depends on the characteristics of extracellular compounds that bind Hg^2+^ in the external environment. In this instance, some substances promote the uptake of mercury ions into the cell and subsequent methylation, while others inhibit both processes [142].

#### 3.3.2. Specific Resistance: *mer* Operon Genes

Research has identified a predominance of mercury resistance at the molecular level in bacteria [143]. The mer operon is the most extensively studied mechanism responsible for mercury inactivation in bacteria. It originally evolved in geothermal bacteria. Recent studies have indicated the presence of the operon in a variety of bacterial samples, including those derived from aerobic, anaerobic, aquatic, and soil environments [144]. The *merB* operon gene plays a pivotal role in the conversion, transport, and detoxification of highly toxic organic compounds like Hg. *merA*, in conjunction with a set of genes encoding membrane transporters, which are typically closely related in the operon, contributes to the deactivation of inorganic compounds [145]. Central to the mer operon is the *merA* gene, which encodes a flavin-dependent NAD(P)-disulfide oxidoreductase (MerA). This enzyme catalyses the conversion of Hg^2+^ to Hg^0^, with the resulting product subsequently diffusing out of the cell [146,147]. The current process is distinct from the majority of other HM detoxification systems in bacteria, which primarily utilise ATPase-based transport systems to facilitate the movement of metal ions across cell membranes or to sequester toxic ions in extracellular structures [148].

**Figure 2:**
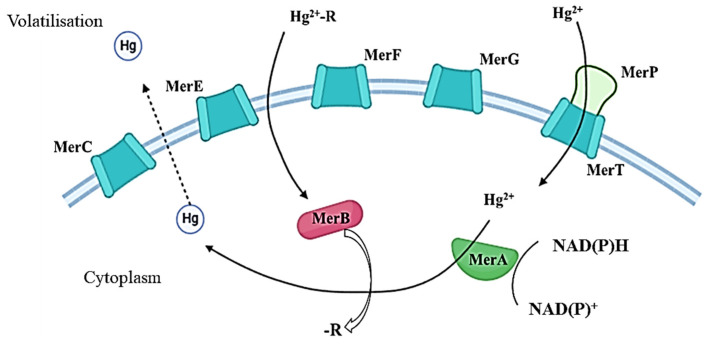
Main pathways of Hg conversion and transport by Mer family proteins. Hg^2+^-R—organic compounds; Hg^2+^—inorganic compounds.

MerA confers a narrow spectrum of resistance to inorganic mercury only. MerB, an organomercury lyase encoded by the *merB* gene, provides a broader spectrum of resistance to mercury. This enzyme catalyses the cleavage of the C–Hg bond in organomercury compounds, thereby enabling the subsequent reduction of Hg^2+^. The operon under consideration also contains several coding genes that are responsible for the periplasmic protein MerP and inner membrane-spanning proteins MerC, MerE, MerF, MerG, and MerT (Figure 2) [143]. The overall gene expression is regulated by MerR and MerD proteins, which function as a promoter and co-repressor of transcription in the presence and absence of Hg²⁺, respectively [144,145,146,147,148,149,150].

In contrast to many aerobic microorganisms, which recover mercury via a specific enzymatic pathway encoded by the mer operon [151], Hg recovery in anaerobic microorganisms occurs through metabolic pathways associated with anaerobic respiration, fermentation, and anoxygenic photosynthesis [152]. Previously [153,154], before the emergence of contemporary methodologies for identifying methylmercury compounds in various media, a diverse array of aerobic and anaerobic Gram-positive and Gram-negative bacteria, as well as fungi capable of producing this compound, were documented. Recent studies utilising pure cultures have documented the capacity to synthesise methylmercury from inorganic compounds exclusively in sulfate-reducing bacteria (predominantly *Desulfovibrio* spp. and *Desulfobulbus* spp.) [135,136,155] and iron-reducing bacteria (predominantly *Geobacter* spp. and *Shewanella* spp.) [137,138,156].

Mercury resistance genes encode proteins involved in the uptake and regulation of mercury, as well as the enzymatic reduction of toxic mercury ions (Hg^2+^) to less toxic elemental mercury (Hg^0^). These processes facilitate mercury detoxification and influence its mobility and bioavailability in the environment. These enzymes facilitate the accelerated conversion of Hg^2+^ and, occasionally, MeHg into elemental metallic mercury (Hg^0^). In 2012, the initial database comprising publicly accessible genomes was assembled. This database encompasses 272 mer operons derived from the genomes of 246 distinct bacterial and archaeal isolates [143,144,157]. Research into *mer* genes continues, and new data have emerged on mer operons in nitrogen-fixing rhizobia. These discoveries have important implications not only for soil bioremediation but also for host plants growing in mercury-contaminated soils [158].

Phylogenetic analysis of *merA* homologues was conducted by Barkay et al. in 2010 [151] and demonstrated that the earliest branching of the *merA* gene originated from thermophilic bacteria (*Aquificales*), whereas in archaea it was acquired by lateral gene transfer. The complexity of the mer operon structure, attributable to the presence of other genes related to *merA* in the operon structure, increased over time during the process of additional genetic information being recruited. The transformation resulted in an improved Hg detoxification apparatus with increased efficiency and function by enhancing the spectrum of mercury organic compound detoxification involving the *merB* gene [143].

*mer* resistance genes can be localised on mobile genetic elements [130]. The strain *Pseudomonas* sp. K-62 harbours plasmids pMR26 and pMR68, which provide broad-spectrum resistance to mercury. These plasmids contain three *mer* gene clusters that confer bacterial resistance to mercury ions and organomercury compounds, encoding the mercury transport system and organomercury lyase [159]. Strain K-62 was isolated from soil contaminated with phenylmercury, exhibiting a tolerance level of 450.0 mg/kg HgCl_2_, 120.0 mg/kg phenylmercury acetate, and 20.0 mg/kg methylmercury phosphate [48].

One group of microorganisms showing great potential for the purpose of bioremediation of contaminated territories is that of *Actinomycetes*. *Actinomycetes* have been shown to possess the capacity for the biodegradation of a wide range of organic and inorganic xenobiotics, as well as a high level of biosorption and bioaccumulation of HM ions. They are identified as playing a dominant role in the processes of natural self-purification in open ecosystems, exhibiting ecological plasticity, and lacking pronounced pathogenic properties [41,76,77,159,160].

It is known that some actinomycete strains tolerant to other HMs form protein complexes that encourage further binding of HMs. These also have ATP-dependent channels that ensure the outflow of toxic HM ions from the cell [76,77,161,162].

A bioinformatic search of the NCBI database (https://www.ncbi.nlm.nih.gov/, accessed on 17 December 2024) was conducted using keywords associated with mercury resistance (e.g., “mercury resistance genes”, “mer operon”, “actinomycetes”, “Hg resistance”) to identify the most prevalent coding sequences that may underlie the resistance of actinomycetes to elevated concentrations of Hg ions in the growth medium. This tolerance is attributed to the conversion of toxic forms to metallic Hg, which is subsequently expelled from the cell. Information regarding encoded proteins can be found on mobile genetic elements and in the chromosome (Table 3).

A bioinformatic search of the NCBI Protein database (https://www.ncbi.nlm.nih.gov/protein/, accessed on 24 December 2024) allows us to identify the most prevalent proteins in *Actinomycetes* representatives. The presence of these proteins is indicative of various forms of resistance, the presence of transport systems, and the increasing accumulation of bacterial cells in Hg(II) ions (Table 4).

As can be observed, the vast majority of Hg resistance proteins and genes are associated with the mer operon, which is consistent with information from the literature.

## 4. Methods of Hg Removal from Pollution Media

### 4.1. Physicochemical Methods

Various chemical methods for the removal of mercury from aqueous solutions have been employed experimentally in research laboratories and industrial settings [163,164,165,166]. However, the application of such methodologies for soil and water purification in natural environments is hindered by several challenges. Primarily, these methods require substantial quantities of chemical reagents, which may lead to secondary contamination of environmental sites. Numerous experimental studies have been conducted on the removal of mercury from aqueous solutions. These include methods such as coagulation, filtration, adsorption by activated carbon, ion exchange, chemical reduction, precipitation, and reverse osmosis [27,75,167]. Physicochemical methods have their advantages and disadvantages (Table 5). The most frequent undesirable consequences are the costliness of the methodology, long-time expenditures, and formation of secondary pollutants. Therefore, there is an urgent need to develop advanced, safe, cost-effective, and efficient technologies for Hg neutralisation.

### 4.2. Biological Objects Perspective for Hg Removal from Contaminated Media

**Microalgae**. Microalgae of various species are able to thrive in industrial wastewater, agricultural wastewater, and domestic wastewater that contain elevated concentrations of inorganic and organic substances. As demonstrated in [172], common HMs in wastewater have a significant detrimental effect on the viability of microalgae. The toxicity of HMs to most microalgae species varies significantly; the relative toxicity series is as follows: Hg > Cd > Cu > Zn > Pb > Co > Cr [173,174].

In the development of methodologies for the treatment of wastewater from HM, the green algae *Chlorella* are frequently utilised (Table 1). In the context of employing *C. vulgaris*, the efficacy of Hg removal at an initial concentration of 10.0 µg/L Hg in the medium is observed to reach 95% when utilising genetically modified cells, in comparison to the performance of native cells [51]. The use of *Chlorella* as a dietary supplement leads to a significant increase in the adsorption of mercury from the human body. In this regard, *Chlorella* has been shown to exhibit considerable potential for metal adsorption, making it a promising candidate for mitigating the toxic effects of mercury compounds [175]. The effective use of microalgae and bacterial consortia has demonstrated significant potential for mercury remediation. For instance, *Chlamydomonas* has been shown to remove up to 75% of the mercury initially present in solution [176]. The detoxification of wastewater using *Chlamydomonas* in combination with bacterial consortia represents a promising strategy for reducing environmental pollutant levels [177,178].

The blue-green alga *Limnothrix planctonica* can transform up to 60.0 µg/kg Hg^2+^ into a form with special properties—β-HgS. Such transformations have also been observed in the green alga *Selenastrum minutum* [54]. Under aerobic conditions, Hg^2+^ is predominantly transformed into β-HgS, a process that occurs in both prokaryotic and eukaryotic algae. This has significant implications for the cycling of Hg compounds in the aquatic environment [54].

**Fungi**. Recent studies (Table 1) have reported on certain species of fungi that contribute to mercury biovolatilisation [60]. This finding suggests that they may play a role in the biogeochemical cycle of Hg and that they have the potential to remediate contaminated soil using biological means.

The precise mechanisms underlying the transformation of mercury by fungi, as well as their resistance and tolerance to Hg, remain to be fully elucidated. This limitation hampers the potential for the widespread application of fungi in the bioremediation of mercury-contaminated environments. In laboratory experiments, certain species of microscopic fungi have been observed to possess the capacity to accumulate and transform toxic forms of mercury. Examples of such species include *Penicillium* sp., *Aspergillus* sp., *Rhizopus* sp., and *Trichoderma* sp. [179]. The strain *Penicillium* spp. DC-F11 was found to have the potential to reduce phytotoxicity of Hg^2+^ and total and exchangeable Hg in soil up to 26% at an initial Hg concentration of 100.0 mg/kg dry weight [60]. As demonstrated in studies [180,181,182], fungi have the capacity to reduce Hg^2+^ toxicity through extracellular sequestration by adsorption and deposition. Primary adaptive intracellular responses to Hg^2+^ exposure have been identified that include *merA* and *merB* homologues [183], metabolism of thiol compounds [184], defence against oxidative stress, and metabolism for damage repair [185]. Apparently, fungal resistance to Hg is a consequence of a multisystem process.

Yeasts have demonstrated significant potential for bioremediation. Due to their high degree of self-aggregation and ability to separate from aqueous media, they can be effectively applied in the biological treatment of Hg-contaminated water. As with other bioremediation agents, different yeast species exhibit varying degrees of resistance to toxicants. A comparison of two yeast species revealed that *Candida albicans* is more tolerant to mercury and can survive at 0.75 µg of HgCl_2_. It also produces significantly higher amounts of methylmercury (13.0 ng/mL) than *Saccharomyces cerevisiae* (6.0 ng/mL) [58].

Fungi are capable of intracellular accumulation and deposition of Hg at the cell growth stage. For instance, the Hg-tolerant yeast *Yarrowia* spp. has been observed to accumulate up to 50% Hg from liquid culture medium at an initial content of 870.0 µg. The majority of the adsorbed Hg is found in the cell wall and spheroplasts. As demonstrated in [63], while there is evidence of trace quantities of Hg being found in the mitochondria, the majority of Hg is found in the nuclei and other organelles of the cell. The aforementioned evidence indicates mercury bioaccumulation.

**Bacteria.** The ability of bacteria to withstand the toxic effects of Hg forms the basis for bacterial remediation of contaminated environments. In order to survive under conditions of mercury stress, microorganisms have developed a large number of adaptations for Hg detoxification [43,144,186]. As previously stated, bacteria and archaea employ the operon to enzymatically reduce Hg^2+^ or MeHg to the volatile, less toxic form, Hg^0^. The development of carriers and consortiums based on Hg-volatilising bacteria is a consequence of the high efficiency of the system [187]. In this process, Hg is removed after reduction to Hg^0^ by evaporation and collection on porous adsorbents [188].

The development of an effective bioremediation approach can be facilitated by leveraging the recovery mechanism activated by the *merA* gene. It is important to note that other detoxification methods also exist, namely biosorption and bioaccumulation. Mercury can be both accumulated and taken up by living bacterial cells as well as by dead bacterial biomass [141].

A wide range of Gram-positive and Gram-negative bacteria (Table 1) have a unique system of tolerance and subsequent conversion of toxic forms of Hg to non-toxic forms. It has been demonstrated that their abundance in the medium can increase with increasing Hg levels [186,189].

As well as the *mer* system, other mechanisms of Hg detoxification have been described. For example, some substances can limit Hg^2+^ entry into cells [95,190]. The binding of Hg^2+^ to the endoplasmic reticulum has been found to provide a low level of bacterial tolerance to this type of stress. It was also determined that thiol compounds and the antioxidant system are involved in the mechanism of resistance exhibited by bacterial cells to Hg [191]. Different bacteria can be used in the bioremediation of mercury. *Escherichia coli* expressing adsorption proteins can remove mercury once the concentration of mercury in a medium reaches a certain level. Subsequent to the adsorption processes, it is possible to remove bacterial cells with adsorbed Hg from the medium by employing various immobilisation strategies [192].

In the development of mercury bioremediation methods, the use of sulfate-reducing bacteria highly adapted to combined exposure to mercury and other heavy metals is promising. As demonstrated in [193], the inorganic reduction of sulfate to H_2_S by these bacteria can lead to a reduction in the toxicity of heavy metal ions. This, in turn, can lead to a change in the valence of toxic and soluble metals, thereby rendering them immobile and biologically inactive. Other species of sulfate-reducing bacteria have been found to be capable of methylation and demethylation of mercury. The genus *Desulfovibrio* is distinguished by its capacity to synthesise MeHg. As demonstrated in [67], the level of production varies between different species within the range of 0.14 to 7.30 nM/L^−1^.

The isolation of Hg-tolerant bacterial strains from soils and water bodies contaminated with HM is of particular concern. α-*Proteobacteria* demonstrate elevated resistance to Hg, with a minimum inhibitory concentration of 33.5 mg/L. The detection of mercury volatiles, in conjunction with the presence of mercury reductase enzyme, provided evidence that *Proteobacteria* have the capacity to absorb Hg [73].

*Bacillus cereus* and *Drepanocladus revolvens* species have a high capacity to adsorb Hg and bioaccumulate up to 67.0 mg mercury/g dry biomass under aqueous conditions [64]. Some aerobic bacteria and archaea, for example, *B. cereus* and *Pseudomonas putida*, have been observed to reduce soluble Hg⁺ ions to elemental Hg^0^ using the MerA protein [194]. At the same time, an operon present on the VS1 plasmid confers resistance to 20.0 μM Hg^2+^ to *P. aeruginosa*, which further promotes reduction processes to metallic Hg [74]. *P. putida* V1 has the ability to convert HgCl_2_ to gaseous Hg^0^ and degrade MeHg, thimerosal, and phenylmercury acetate [75]. Furthermore, the capacity of certain bacterial strains to demethylate methylmercury has been demonstrated. This process significantly reduces the risk of bioaccumulation at subsequent parts of the food chain [195].

Cyanobacteria are also involved in Hg^2+^ biotransformation processes. Analyses have revealed the presence of free Hg^2+^ ions and their complexes, as well as metacinnabar (β-HgS), which constitutes the main biotransformed mercury pool associated with bacterial cells. Such products have been detected during transformation in species such as *Limnothrix planctonica* (Lemm.), *Synechococcus leopoldiensis* (Racib.) Komárek, and *Phormidium limnetica* (Lemm.). In the initial phases of Hg exposure, there is a rapid synthesis of β-HgS and Hg^0^ among the representatives. The available data suggest that cyanobacteria located at the water-air interface are capable of converting substantial quantities of Hg^2+^ into β-HgS within the environment [71].

Furthermore, the presence of aerobic bacteria has been demonstrated to facilitate the detoxification of both inorganic Hg and methylmercury compounds. For instance, the soil aerobic nitrogen-fixing bacterium *Xanthobacter autotrophicus* exhibits a tolerance to 0.04 mg/L^−1^ Hg^2+^ and 0.01 mg/L^−1^ MeHg. The bacterium has been shown to be capable of removing mercury compounds from the medium through a process of reduction and volatilization of Hg^0^ [78].

Toxic metal exposure can harm microbial communities in water and soil, hinder the remediation of contaminated environments, and promote the proliferation of antibiotic-resistant pathogens [196,197]. In this regard, the study of mercury tolerance in the main antibiotic producers is of extreme relevance. Among the *Streptomyces* representatives, strains exhibiting resistance to HgCl_2_ at a concentration of 1.0 mM and phenylmercury acetate have been identified [198,199]. The study, along with others in the field, corroborates the emergence of tolerance as a consequence of the prolonged impact of HM on soil microbiota, in conjunction with the selection of resistance to antibiotics [200,201].

It is imperative to study representatives of deep-sea bacteria due to the fact that the process of Hg methylation occurs under oxygen-free conditions at the bottom of water bodies. For instance, strains of *P. stutzeri*, *Bacillus* sp., and *Pseudoalteromonas* sp. isolated from the central Indian Ocean have increased tolerance to mercury compounds up to 100.0 mg/L as HgCl_2_ and a high potential for Hg removal from the medium. In the context of a laboratory setting, the isolates have been observed to demonstrate the capacity to eliminate up to 80% of HgCl_2_ from a culture fluid sample with an initial HgCl_2_ content of 50.0 mg/L Hg^2+^ [65].

Actinomycetes of the genus *Rhodococcus* represent a group of microorganisms with considerable potential for remediation of contaminated areas affected by mercury pollution. As demonstrated in [76], tolerance to mercury in *R. erythropolis* BD2 is attributable to the location of mercury resistance genes on the plasmid that carries the mer operon [202]. The loss of this plasmid consequently results in the loss of bacterial resistance to Hg ions. Another tolerant strain, *R. qingshengii* RL1, has been identified relatively recently [77]. *R. qingshengii* RL1 has been shown to be capable of survival at an Hg concentration of 1.0 mM in nutrient medium [77]. The presence of mercury resistance genes has been identified in this strain, including the coding sequences of the MerR transcription regulator located in the chromosome and a unique alkyl-mercury lyase involved in the degradation of toxic organomercury compounds [77,203].

Some bacterial strains exhibit characteristics of polyresistance to common toxic metals, including cadmium, lead, mercury, and arsenic. The *E. coli* K-12 strain, which produces the periplasmic protein ZinT, has been demonstrated to bind Ni, Zn, Cd, and Hg and to convert them into less toxic forms [204,205]. In this study, bacterial isolates of *Enterobacteriaceae* were obtained from the water of Bo Ismail Bay in Algeria. These isolates were found to demonstrate tolerance to several heavy metals, including Zn, Cu, Pb, Hg, and Cd. Concurrently with HM resistance, strains demonstrate tolerance to amoxicillin, imipenem, cefotaxime, tetracycline, gentamicin, ciprofloxacin, and trimethoprim-sulfamethoxazole, thereby substantiating the combined resistance of bacteria to diverse toxicants [69]. Nonetheless, the presence of multiple HM ions in the medium gives rise to a competitive interaction with reactive groups on the cell surface [206]. The uptake of Hg can be significantly (more than 50%) reduced in the presence of competing HMs, such as Zn, Cd, and Ni [207]. Research indicates that Hg-methylating bacteria possess the ability to generate methyl lead and methyl cadmium, which are classified as highly toxic organic compounds [208].

These are not all the known representatives of microorganisms that could potentially be used in the development of eco-innovative methods for the remediation and detoxification of mercury wastes from industrial and coastal effluents, as well as from the natural environment. Further research is required to identify the precise molecular mechanisms underlying enhanced mercury tolerance and to optimise growth conditions to maximise the predicted mercury removal potential.

## 5. Conclusions

There is a long-standing and profoundly serious concern regarding the presence of mercury compounds within open ecosystems. In the aftermath of large-scale environmental disasters, mercury pollution has emerged as a significant threat to living organisms. The heightened toxicity of this pollutant poses a grave and enduring risk to human health and the integrity of the biosphere. The presence of both organic and inorganic Hg compounds in the environment, even in trace amounts, creates the risk of chronic toxic effects on living organisms and threatens the stability of natural ecosystems. It is evident that traditional physical and chemical methods for combating this toxic substance are ineffective and environmentally unsafe. Consequently, researchers are actively developing biotechnological approaches. These approaches utilise active microbial strains or consortia to enhance the detoxification and degradation of pollutants in the environment. These microorganisms possess the capacity to effectively detoxify and inactivate ecopollutants in aquatic and terrestrial ecosystems. An increasing body of research focuses on the fundamental study of mercury’s bioavailability and toxic effects on natural microbial communities. These communities play a primary role in responding to pollutants and accumulating hazardous substances. A comprehensive investigation into the mechanisms of detoxification, biodegradation, and bioremediation of Hg by new strains of bioaccumulators, accompanied by detailed physiological and biochemical characterisation, will facilitate the development of innovative technical solutions for the remediation of contaminated sites. This research will also contribute to the development of environmentally safe biotechnological approaches for the neutralisation and utilisation of hazardous ecotoxicants in the near future. Looking ahead, it is important to prioritise research on the genetic and metabolic enhancement of mercury-resistant microorganisms to improve their detoxification potential. Further studies on microbial community dynamics, horizontal gene transfer, and the development of molecular tools for real-time monitoring will deepen understanding of mercury resistance and transformation. Integration of comprehensive analyses and pilot-scale field studies will be essential to validate and implement effective bioremediation strategies. Combining microbial bioremediation with phytoremediation and physicochemical methods may offer more comprehensive and sustainable solutions for mercury pollution.

## Figures and Tables

**Table 1:** Source of isolation and species composition of Hg-accumulating microorganisms.

Organism	Resistance	Sample Characteristics/ Sampling Location	References
**Microalgae**			
*Chlorella* sp.	2.89 mg/L	Live biomass of *Chlorella* sp. immobilised on dry fruits of *Luffa cylindrical*, Universidad de Sucre, Cinselejo, Colombia	[49]
*Ch. sorokiniana* CCAP 211/8K	5.0 mg/L	The Culture Collection of Algae, University of Texas at Austin (UTEX), Austin, TX, USA	[50]
*Ch. vulgaris*	0.0095 mg/L	Freshwater, Institute of Hydrobiology, Chinese Academy of Sciences, Wuhan, Hubei, People’s Republic of China (PRC)	[51]
*Chlamydomonas reinhardtii* wild-type CC-125	1.904 mg/L	Chlamydomonas Resource Center, University of Minnesota, St. Paul, MN, USA	[52]
*C. reinhardtii*	0.000666 mg/L	Culture Collection of Algae at the University of Texas at Austin, UTEX	[53]
*Limnothrix planctonica*	0.06 mg/kg dry weight	Leaf surface of *Nuphar variegatum*, Tasso Lake, Ontario, Canada	[54]
*Chlorococcum dorsiventrale* Ch-UB5	2.630 mg/L	Bentos, Mahdia Coast, Republic of Tunisia	[55]
*Scenedesmus* sp.	0.004 mg/L	Seawater, Ozogoche Lagoon, Sangay Lagoon National Park, Republic of Ecuador	[56]
*S. obtusus* XJ-15	95.0 mg/L	Hubei Key Laboratory of Mineral Resources Processing and Environment, Wuhan University of Technology, Wuhan, PRC	[57]
*Pleurococcus* sp.	0.006 mg/L	Sucus Lagoon, Cayambe-Coca Ecological Reserve, Republic of Ecuador	[56]
**Fungi**			
*Candida albicans*	0.75 mg/L	Collection strain, Department of Microbiology and Molecular Genetics, Technion-Israel Institute of Technology, Haifa, State of Israel	[58]
*Mucor hiemalis* EH 8	50.0 mg/L	Microbial biofilm in sulphide-reduced spring water, Marching site, Bavaria, Federal Republic of Germany	[59]
*Penicillium* spp. DC-F11	74.0 mg/kg dry weight	Soil contaminated with heavy metals, PRC	[60]
*Rhodotorula mucilaginosa* TR52	58.0 mg/L	Water from Caño Amarillal, Amazon forest, Vaupés department, Republic of Colombia	[61]
*Saccharomyces cerevisiae* PTCC 5010	0.005 mg/L	Lyophilised culture, Persian Type Culture Collection, Tehran, Islamic Republic of Iran	[62]
*Yarrowia* spp. Idd1	32.0 mg/kg dry weight	Seawater, Lagos Lagoon, Nigeria	[63]
*Yarrowia* spp. Idd2	59.0 mg/kg dry weight	Seawater, Lagos Lagoon, Nigeria	[63]
*Y. lipolytica* TR55	51.0 mg/L	Water from Caño Amarillal, Amazon forest, Vaupés Department, Republic of Colombia	[61]
**Bacteria**			
*Bacillus* sp.	100.0 mg/L	Seawater, central Indian Ocean	[64]
*B. cereus* MTCC 10650	67,000.0 mg/kg dry weight	Soil. Microbial Type Culture Collection and Gene Bank (MTCC), housed at the Institute of Microbial Technology (IMTECH), Chandigarh, Republic of India	[65]
*Burkholderia contaminans* TR100	71.0 mg/L	Sediment from the Caño Rojo Creek, Amazon forest, Republic of Colombia	[61]
*Desulfobulbus propionicus MUD DSMZ 6523*	0.0007 mg/L (MeHg)	German Collection of Microorganisms and Cell Cultures of the Leibniz Institute (DSMZ), Braunschweig, Federal Republic of Germany	[66]
*Desulfovibrio desulfuricans* ND132	0.00014–0.0073 mg/L	DSMZ, Braunschweig, Federal Republic of Germany	[67]
*D. africanus* DSMZ 2603	0.00045 mg/L (MeHg)	DSMZ, Braunschweig, Federal Republic of Germany	[66]
*Ensifer medicae * AMp10	0.25 mg/L	*Medicago polymorpha* rhizosphere, Almadén mining district, Ciudad Real, Spain	[68]
*Citrobacter freundii*	200.0 mg/L	Seawater, Bou Ismail Gulf, Algeria	[69]
*Klebsiella* sp. BacI31	125.0 mg/L	Aeschynomene luminensis rhizosphere, wetland biome, mercury contaminated soil (Hg concentration up to 3.24 mg/kg), Federative Republic of Brazil	[70]
*Limnothrix planctonica* (Lemm.)	3200.0 mg/L	*Nuphar variegatum* leaf surface, Tasso Lake, Lake of the Bays, Ontario, Canada	[71]
*Photobacterium* spp. MELD1	33.0 mg/kg dry weight	Mercury and dioxin heavily contaminated rhizosphere soils of reed *Phragmites australis* at An-shun factory site, Taiwan	[72]
*Phormidium limnetica* (Lemm.)	3200.0 mg/L	Water from Lake Opinicon, Ontario, Canada	[71]
*Sphingopyxis* sp. SE2	33.5 mg/L	Soil, New South Wales, Australia	[73]
*Pseudoalteromonas* sp.	100.0 mg/L	Seawater, central Indian Ocean	[65]
*Ps. stutzeri*	100.0 mg/L	Seawater, central Indian Ocean	[65]
*Pseudomonas* sp. TP30	64.0 mg/L	Sediment, Lake Tipiska, Amazon forest, Amazon department, Republic of Colombia	[61]
*P. aeruginosa* FA-2	5.44 mg/L	Sanitary landfill, Keputih district, Sukolilo district, Madura Strait coastal area, Indonesia	[74]
*Pseudomonas monteilii* BacI6	125.0 mg/L	*Polygonum acuminatum* rhizosphere, wetland biome, mercury contaminated soil (Hg concentration up to 3.24 mg/kg), Federative Republic of Brazil	[70]
*P. putida* V1	2.37 mg/L	Soil, State of Rio Grande do Sul, Brazil	[75]
*Rhizobium leguminosarum* bv. *trifolii* STf07	0.068 mg/L	*Trifolium fragiferum* rhizosphere, Almadén mining district, Ciudad Real, Spain	[68]
*Rhodococcus erythropolis* BD2	816.0 mg/L	Institute of Microbiology, Georg August University, Göttingen, Germany	[76]
*R. qingshengii* RL1	272.0 mg/L	Extracted from the leaves of *Eruca sativa* L., Germany	[77]
*Xanthobacter autotrophicus*	0.04 mg/L Hg^2+^, 0.01 mg/L MeHg	American Type Culture Collection (ATCC), USA	[78]

**Table 3:** The most prevalent mercury resistance genes in *Actinomycetes*.

No	Gene Symbol	Product	Locus Tag	Organism	Location of the Coding Sequence	Gene Length, nt	Protein Length, aa
1	*merA*	Mercury (II) reductase	C5O27_RS02895	*Gordonia alkanivorans* YC-RL2	Chromosome Complement (618,730–620,151)	1422	473
			BCM27_RS13490	*G. terrae* 3612	Chromosome Complement (2,996,454–2,997,890)	1437	478
			IHQ52_RS14160	*G. amicalis* CEGA1	Chromosome Complement (3,090,014–3,091,450)	1437	478
			ABWI03_RS31730	*Rhodococcus baikonurensis* NPDC097667	Chromosome Complement (9129–10,565)	1437	478
			F1734_RS25830	*R. ruber* C1	Plasmid: unnamed1 129,228–130,664	1437	478
			VYL96_RS16955	*Dietzia cinnamea* 55	Plasmid: unnamed 18,650–20,050	1401	466
			VYL96_RS00490	*D. cinnamea* 55	Chromosome Complement (111,188–112,582)	1395	464
			JOE55_RS10340	*Kocuria palustris* TAGA27	Chromosome Complement (2,325,179–2,326,603)	1425	584
			O4162_RS16800	*D. maris* ИЭГM 44	Chromosome Complement (5029–6429)	1401	466
2	H351_RS26335	MerR family transcription regulator	H351_RS26335	*R. erythropolis* R138	Chromosome 5,724,143–5,724,568	426	141
3	NFA_RS29315	MerR family DNA-binding protein	NFA_RS29315	*Nocardia farcinica* IFM 10152	Plasmid: pNF2 58,358–58,759	402	341
4	Y013_RS03165	DNA-binding transcriptional regulator of the MerR family	Y013_RS03165	*R. pyridinivorans* SB3094	Chromosome 679,626–680,465	840	279
5	NFA_RS28365	P-type cation-translocating ATPase	NFA_RS28365	*N. farcinica* IFM 10152	Plasmid: pNF1 39,781–41,745	1965	654
6	*merH*	Hg^2+^ MerH transporter	MAB_RS00055	*Mycobacteroides abscessus* ATCC 19977	Plasmid: pMAB23 5371–5883	513	170
7	CEQ30_RS22845	Protein containing DUF3817 domain	CEQ30_RS22845	*N. brasiliensis* FDAARGOS_352	Chromosome 5,069,600–5,069,899	300	99
8	MAB_RS00040	MerT mercury transporter	MAB_RS00040	*Myc. abscessus* ATCC 19977	Plasmid: pMAB23 Complement 2964–3398	435	144
9	Spa2297_RS28755	Mercury reductase	Spa2297_RS28755	*Streptomyces parvulus* 2297	Chromosome 6,398,621–6,400,009	1389	462

**Table 4:** The most prevalent proteins that determine Hg resistance in *Actinomycetes*.

No	Locus	Region Name	Definition	Organism	Protein Length, aa
1	PZT88875	TrxA	MAG: Mercury vector (Gram-positive bacteria with high G + C content)	*Gordonia* sp.	172
2	OLT52902	TrxA	Mercury transporter	*Gordonia* sp. CNJ-863	171
3	OBA35129	CcdA	Mercury transporter	*Gordonia* sp. 852002-51296_SCH5728562-b	301
4	OZC38182	TrxA	Mercury transporter	*Rhodococcoides fascians*	174
5	WP_283289544	PRK13239	Organomercurial lyase MerB	*Microbacterium* sp.	218
6	WP_367652617	PRK13239	Organomercurial lyase MerB	*R. pyridinivorans*	278
7	WP_374610375	–	Organomercurial lyase MerB (in Gram-positive bacteria with high G + C content)	*Gordonia* sp.	213
8	WP_231381052	HTH_MerR-SF	Organomercurial lyase MerB	*G. alkanivorans*	373
9	ATD71119	PRK13239	Alkylmercury lyase	*Gordonia* sp. 1D	216
10	KSU68309	PRK13239	Alkylmercury lyase	*R. qingshengii*	222
11	ANY23673	PRK13239	Alkylmercury lyase MerB	*G. terrae*	216
12	ORC17824	PRK13239	Alkylmercury lyase	*R. qingshengii*	218
13	GGB48061	ArsR	Repressor of the mercury resistance operon MerR	*G. jinhuaensis*	125
14	CCW11083	Haloacid Dehalogenase-like Hydrolases	ATPase that transports lead, cadmium, zinc, and mercury	*R. aetherivorans*	321
15	CCW11076	TrxA	Mercury resistance operon ORF3 (precursor)	*R. aetherivorans*	181
16	AWK76739	TrxA	Mercury transporter (plasmid)	*R. oxybenzonivorans*	171
17	AXY49768	TrxA	Mercury transporter	*R. ruber*	179
18	BAX98527	CcdA	Mercury resistance transport protein/cytochrome C biogenesis protein	*Myc. stephanolepidis*	236
19	WP_371955153	–	Mercury resistance system, periplasmic binding protein MerP	*Actinomadura* sp. DLS-62	82
20	EGQ75422	CopZ	MerTP family, mercury (Hg^2+^) permease, MerP binding protein	*Actinomyces* sp.	87

**Table 5:** Advantages and disadvantages of physicochemical methods of Hg removal from aqueous solutions.

Method	Advantages	Disadvantages	Removal Mechanism	References
Ion exchange	Removes many contaminants, has high efficiency	It is imperative that ion exchange resins are periodically maintained, a process which is time-consuming	Reversible chemical reaction	[168]
Precipitation	Characterised by ease of operation, cost-effectiveness, and high selectivity.	Production of large quantities of sludge	Chemical reaction	[169,170]
Membrane filtration (Reverse Osmosis, Electrodialysis, Nanofiltration)	High efficiency, production of less toxic waste, and removal of multi-component pollutants	Expensive method and maintenance, production of waste, loss of huge amounts of water	Selective membrane permeability	[171]
Adsorption	The use of raw materials that are both inexpensive and highly efficient, as well as simple operation, is a key advantage	High cost of sorbent regeneration, different uncontrolled removal capacity of adsorbents	Physical or chemical adsorption	[3]

## Data Availability

No new data were created or analyzed in this study. Data sharing is not applicable to this article.

## Abbreviations

The following abbreviations are used in this manuscript:

WHO	World Health Organization
HM	Heavy metal
Hg	Mercury
As	Arsenic
Cd	Cadmium
Pb	Lead
Zn	Zinc
DNA	Deoxyribonucleic acid
MeHg	Organomercury/monomethyl
ROS	Reactive oxygen species
EPS	Extracellular polymeric substances
LPS	Particularly lipopolysaccharides
EPSs	Exopolysaccharides
RNS	Reactive nitrogen species
RNA	Ribonucleic acid
NCBI	National Center for Biotechnology Information
